# Berberine-Loaded Chitosan-Succinylated Pullulan Composite Films for the Preservation of Fresh-Cut Apples

**DOI:** 10.3390/polym18080908

**Published:** 2026-04-08

**Authors:** Xinyu Zhang, Chu Gong, Yujie Liu, Jun Wang, Zhizhou Yang, Jun-Li Yang

**Affiliations:** 1 School of Materials Science and Engineering, Qilu University of Technology (Shandong Academy of Sciences), Jinan 250353, China; 19863069998@163.com; 2 Shandong Laboratory of Advanced Materials and Green Manufacturing at Yantai, Yantai 264006, China; gongchu@amgm.ac.cn (C.G.); 18435996332@163.com (Y.L.); licpwangjun@163.com (J.W.); 3 CAS Key Laboratory of Chemistry of Northwestern Plant Resources and Key Laboratory for Natural Medicine of Gansu Province, Lanzhou Institute of Chemical Physics (LICP), Chinese Academy of Sciences (CAS), Lanzhou 730000, China

**Keywords:** chitosan, succinylated pullulan, berberine, films, preservation properties

## Abstract

Biopolymer-based packaging films possess outstanding performances and are being developed as the alternatives to traditional petroleum-based plastic packaging films with many non-ignorable shortcomings. In this study, chitosan, succinylated pullulan (SP), and berberine (BBR) were combined to fabricate novel biopolymer-based composite films (CSSPB) via the layer-by-layer assembly method. The effects of the incorporation of BBR on the physicochemical properties of the film were investigated. It was found that after BBR was added, the tensile strength (TS), elongation at break (EAB), hydrophobicity, and antioxidant capacities of the film were enhanced. The chemical bonding, crystalline properties, elemental composition, and thermal stability of the films were also characterized by Fourier transform infrared (FT-IR) spectroscopy, X-ray diffraction (XRD), X-ray photoelectron spectroscopy (XPS), and thermogravimetric analysis (TGA), respectively. The in vitro antifungal tests revealed the antifungal activities of the films with a relatively high BBR content against *Colletotrichum gloeosporioides* (CG). In the preservation experiments, the CSSPB films exhibited preservation effects on fresh-cut apples, which manifested as delaying browning, weight loss, an increase in the soluble solids content, and a decrease in hardness. The new CSSPB composite films were opined to hold application potential in the field of food packaging.

## 1. Introduction

Traditional petroleum-based plastic packaging films have advantages such as low cost and high transparency, and have been extensively utilized. However, they are in possession of non-negligible shortcomings, which include but are not limited to the non-biodegradability [1] leading to severe environmental pollution, the lack of antimicrobial activities, and the safety hazard caused by the migration of plastic additives. Researchers are developing biopolymer-based packaging films [2] with excellent performances to replace petroleum-based plastic ones.

Natural polysaccharides as a category of biopolymer can be employed to fabricate biopolymer-based packaging films [3]. Chitosan, the second most abundant biopolymer in nature, is a linear cationic polysaccharide consisting of D-glucosamine and N-acetyl-D-glucosamine units. It has a variety of properties such as film-forming properties, antimicrobial activities, non-toxicity, biocompatibility, and biodegradability [4,5,6]. The application fields of chitosan include agriculture, food, and medicine [7]. Chitosan-based films have been fabricated using many methods such as casting and layer-by-layer, and they can exert antimicrobial activities, barrier properties, and sensing properties [8]. Chitosan-based films have already been applied in the field of food packaging. For example, Liang et al. [9] prepared composite films by the formation of Schiff-base imine bonds between the amino groups of chitosan and the aldehyde groups of oxidized fucoidan, and encapsulating cinnamaldehyde. The physicochemical properties of the films were studied, and the pH-responsive antimicrobial activities were discovered. The films were observed to be capable of extending the shelf life of litchi fruits. Pan and co-workers [10] synthesized an antifungal chitosan derivative modified with quaternary ammonium salt and quaternary phosphonium salt, and then employed it along with polyvinyl alcohol to prepare composite films. They researched the effects of the ratio of the derivative to polyvinyl alcohol on the structure and physical properties of films. They observed that the novel composite films were able to effectively preserve mangoes and papayas.

Pullulan is a natural linear polysaccharide produced by microorganisms, possessing numerous intriguing characteristics including good water solubility, film-forming properties, structural modifiability, non-toxicity, non-immunogenicity, non-mutagenicity, and edibility [11,12,13]. It has been widely applied in the biomedical and pharmaceutical field, food industry, cosmetic industry, etc. [14,15]. Pullulan-based films are transparent, flexible, and high-oxygen barrier materials, and they show preservative effects on fruits and vegetables [16]. For instance, Yan and co-workers [17] blended Konjac glucomannan and pullulan to develop composite films, and investigated their physicochemical properties. They also applied the films in the strawberry preservation and found that when the polysaccharide concentration was 1% (*w*/*v*) and the mass ratio of Konjac glucomannan to pullulan was 2:1, the prepared composite film exhibited the best preservation effects on strawberries, especially at 4 ± 1 °C and 85 ± 5% relative humidity. Zhang et al. [18] synthesized D-arginine-succinic anhydride-pullulan with antibacterial activities and used it to fabricate an antibacterial film. The transparency, water solubility, and water vapor permeability of the film were assessed. The film was observed to have a significant preservative effect on cherries.

The isoquinoline alkaloid berberine (BBR) with ultraviolet shielding abilities [19], antioxidant capabilities [20,21], broad-spectrum antibacterial activities [22], and non-toxicity [23], as a natural additive, has been adopted to develop biopolymer-based packaging films. For instance, Öztürk et al. [21] utilized 2-hydroxypropyl-β-cyclodextrin to enhance the water solubility of BBR, and then combined the BBR solution with chitosan to yield a novel film. The introduction of BBR was found to enhance the mechanical, antioxidant, and antimicrobial properties of films. The novel chitosan film containing BBR is promising to be applied in the food packaging industry. Chen and co-workers [24] encapsulated BBR into β-cyclodextrin to obtain the inclusion complex with aggregation-induced emission properties, and then combined it with polyvinyl alcohol to prepare novel films. The films possessed high transparency in the visible range, good biodegradability with the water absorption of 76% and water solubility of 31%, and good mechanical properties. Under irradiation (14.4 J/cm^2^), the films were able to generate reactive oxygen species to kill ~4 log CFU/mL of *Listeria monocytogenes* and *Vibrio parahaemolyticus*. Moreover, the films exhibited preservative effects on salmon fillets. This work supplied new insights for developing green antimicrobial packaging. Given the above, we believe that via the organic integration of chitosan, pullulan, and BBR, new biopolymer-based packaging films with excellent performances can be developed.

In this study, first, the succinylated pullulan (SP) was synthesized through the nucleophilic substitution reaction, and then the novel chitosan-SP-BBR composite films (CSSPB) were prepared using the layer-by-layer assembly method. The physicochemical properties of the films were characterized, which included the transparency, mechanical properties, barrier properties, thermal stability, and antioxidant properties. The in vitro antifungal activities of the films against *Colletotrichum gloeosporioides* (CG) were evaluated by the agar diffusion method. Finally, the preservation effects of the films on fresh-cut apples were investigated. The experimental results suggested that the new composite films demonstrated various satisfactory performances, and could potentially be utilized as food packaging materials.

## 2. Materials and Methods

### 2.1. Materials and Reagents

Pullulan (M_n_ = 73,600 g/mol), succinic anhydride (SA, 99%), 4-dimethylaminopyridine (DMAP, 99%), anhydrous dimethylsulfoxide, chitosan (η = 200–400 mPa·s, deacetylation degree = 84.39%), and 1,1-diphenyl-2-picrylhydrazyl (DPPH, ≥97%) were provided by Shanghai Aladdin Biochemical Technology Co., Ltd. (Shanghai, China). Concentrated hydrochloric acid (36–38%) and glycerol (AR) were obtained from Sinopharm Chemical Reagent Co., Ltd. (Shanghai, China). BBR was supplied by BioBioPha Co., Ltd. (Kunming, China). Acetic acid (AR) and sodium bicarbonate (≥99.5%) were purchased from Shanghai Macklin Biochemical Co., Ltd. (Shanghai, China).

### 2.2. Synthesis of SP

SP was synthesized according to our previous study [25]. Pullulan (5 g), SA (1.25 g), and DMAP (0.153 g) were dissolved in anhydrous dimethyl sulfoxide separately, and then the three resulting solutions were mixed. The obtained mixture was stirred at 50 °C for 24 h. After the completion of the reaction, 110 μL of concentrated hydrochloric acid was added. A dialysis bag with a molecular weight cut-off of 3500 Da was used to dialyze the reaction mixture against deionized water. Finally, the dialyzed solution was freeze-dried to yield the product SP.

### 2.3. Fabrication of CSSPB Films

The CSSPB films were fabricated using the layer-by-layer assembly method. 0.2 g of chitosan, a certain amount of BBR, and 20 mL of 0.3% (*w*/*w*) glycerol aqueous solution were mixed, followed by the addition of 0.2 mL of acetic acid to prepare the chitosan solution. Then, 0.2 g of SP, 0.033 g of sodium bicarbonate, and a certain amount of BBR were dissolved in 20 mL of 0.3% (*w*/*w*) glycerol aqueous solution to obtain the SP solution. The chitosan solution was poured into a polytetrafluoroethylene mold (100 mm × 100 mm × 5 mm) and dried in an oven at 50 °C for 3 h to produce the chitosan film. The SP solution was poured onto the chitosan film and dried in an oven at 70 °C for 3 h. After drying, the composite film that had formed was peeled off from the mold and stored for future use. The film was named CSSPB-x, where x = the total mass of BBR × 100%/the mass of chitosan and SP.

### 2.4. ^1^H Nuclear Magnetic Resonance (NMR) Spectra

The ^1^H NMR spectra of pullulan and SP were determined by an NMR spectrometer (Bruker Ascend 500 MHz) using D_2_O as the solvent to analyze the chemical structures of pullulan and SP.

### 2.5. Light Transmittance of Films

An ultraviolet-visible (UV–Vis) spectrophotometer (Shimadzu, Kyoto, Japan, UV-1900i) was used to measure the light transmittance of the film in the wavelength range of 200 nm to 500 nm.

### 2.6. Film Thickness

The thickness of the film was measured by a micrometer (Deli Group Co., Ltd., Ningbo, China, DL321025B). Ten different positions on the film were randomly selected for thickness measurement, and the average of these measurements was the film thickness.

### 2.7. Micromorphology of Films

The film was cut into small pieces that were suitable for testing and fixed onto the sample stage using a conductive adhesive tape. After the film surface was gold-sputtered, its micromorphology was observed using a scanning electron microscope (SEM) (Tescan VEGA, Brno, Czech Republic).

### 2.8. Mechanical Properties of Films

The tensile strength (TS) and elongation at break (EAB) of the film strip (10 mm × 40 mm) were measured using a universal material testing machine (Shimadzu, Kyoto, Japan, AGS-X 500N) at a tensile rate of 5 mm/min.

### 2.9. Moisture Content (MC) of Films

The initial mass (M_1_) of the film (1 cm × 1 cm) was recorded, and the sample was dried in an oven at 100 °C to a constant weight (M_2_). The MC of the film was calculated according to the following formula:

(1) $$\mathrm{MC}\;=\;\frac{M_{1}-M_{2}}{M_{1}}\times 100\%$$

### 2.10. Water Contact Angle (WCA) of Films

The film sample (2 cm × 2 cm) was prepared, and its WCA was determined at room temperature using a contact angle meter (Biolin Scientific, Gothenburg, Sweden, Theta Flex), via the sessile drop method.

### 2.11. Water Vapor Permeability (WVP) of Films

A 50 mL centrifuge tube containing 3 g of anhydrous calcium chloride was sealed with the film and then placed in a closed environment with a temperature of 23 °C and a relative humidity of 100%. The mass of the centrifuge tube was measured at one-hour intervals. The WVP of the film was calculated according to the following formula:

(2) $$\mathrm{WVP}\;=\;\frac{\Delta W\cdot d}{A\cdot \Delta t\cdot \Delta p}$$

where ΔW was the mass change in the centrifuge tube, d was the film thickness, A was the area of the film, Δt was the time interval, and Δp was the difference in water vapor pressure between the two sides of the film.

### 2.12. Fourier Transform Infrared (FT-IR) Spectra

The FT-IR spectra of pullulan, SP, and the films in the range of 4000–600 cm^−1^ were acquired on an FT-IR spectrometer (Shimadzu, Kyoto, Japan, IR Tracer-100), using the attenuated total reflection (ATR) method.

### 2.13. X-Ray Diffraction (XRD)

The crystalline properties of the film were investigated using an X-ray diffractometer (Bruker, Billerica, MA, USA, D8 ADVANCE). The XRD patterns were obtained under the following conditions: X-ray source: Cu Kα radiation, 2θ range: 5° to 90°, scanning speed: 5°/min.

### 2.14. X-Ray Photoelectron Spectroscopy (XPS)

The elemental composition of the film was analyzed using an X-ray photoelectron spectrometer (Thermo Scientific, Waltham, MA, USA, K-Alpha). The spectra were acquired under the following conditions: X-ray source: Al Kα radiation, spot diameter: 400 μm, pass energy: 100 eV, step size: 1.00 eV.

### 2.15. Thermogravimetric Analysis (TGA)

The thermal stability of the film was assessed using a TG analyzer (Linseis, Selb, Germany, TGA PT1000) under the following conditions: atmosphere: nitrogen, temperature range: 30–500 °C, heating rate: 10 °C/min.

### 2.16. DPPH Radical Scavenging Assay

The antioxidant capacities of the film were determined by the DPPH radical scavenging method. In the experimental group, 1 mL of ethanol, the film (1 cm × 1 cm), and 2 mL of 0.1 mmol/L DPPH ethanol solution were mixed. In the control group, 1 mL of ethanol was mixed with 2 mL of 0.1 mmol/L DPPH ethanol solution. After incubation in the dark for 30 min, the absorbance at 517 nm was measured using a UV–Vis spectrophotometer (Shimadzu, Kyoto, Japan, UV-1900i). The DPPH radical scavenging rate was calculated according to the following formula:

(3) $$\mathrm{DPPH}\;\mathrm{radical}\;\mathrm{scavenging}\;\mathrm{rate}\;=\;\frac{A_{0}-A_{1}}{A_{0}}\times 100\%$$

where A_0_ and A_1_ were the absorbance at 517 nm of the control group and the experimental group, respectively.

### 2.17. In Vitro Antifungal Activities of Films

The in vitro antifungal activities of the film were evaluated using the agar diffusion method. The model fungus was CG (CFCC 82113) obtained from the China Forestry Culture Collection Center. A fungal spore suspension (10^6^ spores/mL) was spread on Potato Dextrose Agar (PDA) medium, onto which a 20 mm diameter film disk was then placed. After incubation at 28 °C for 120 h, the mycelial growth was observed to evaluate the in vitro antifungal activities of the film.

### 2.18. Fruit Preservation

The preservation effects of the CSSPB films were evaluated using fresh-cut apples. A total of 180 uniform apple slices were prepared from fresh and firm apples of the same cultivar, randomly divided into five groups, and covered with the CSSPB films. The apple slices exposed to air without any covering served as the control group. All samples were stored under ambient conditions, and on days 0, 1, 2, and 3, the films were removed, and the browning index (BI), weight loss rate, soluble solids content, and hardness were systematically recorded. Three measurements were performed for each of the parameters above.

#### 2.18.1. BI

The surface color of the fresh-cut apples was measured using a colorimeter (3nh, Guangzhou, China, NR4501). The BI was calculated according to the following formula:

(4) $$\mathrm{BI}\;=\;\frac{\frac{\;a\;+\;1.75L}{5.645L\;+\;a\;-\;3.012b}-0.31}{0.172}\times 100$$

where L represented brightness, a represented the red-green value, and b represented the yellow-blue value.

#### 2.18.2. Weight Loss Rate

The weight of the fresh-cut apples was measured on days 0, 1, 2, and 3. The weight loss rate was calculated using the following formula:

(5) $$\mathrm{Weight}\;\mathrm{loss}\;\mathrm{rate}\;=\;\frac{m_{0}-m}{m_{0}}\times 100\%$$

where m_0_ and m were the weight on days 0 and n, respectively.

#### 2.18.3. Soluble Solids Content

The soluble solids content of the fresh-cut apples was measured using a refractometer (DAIKELI, Chizhou, China).

#### 2.18.4. Hardness

The hardness of the fresh-cut apples was determined using a fruit hardness tester (HANDPI, Yueqing, China, GY-3).

### 2.19. Statistical Analysis

The presented data are shown as mean ± standard deviations (SD), and the comparison between different groups was conducted using one-way ANOVA. A value of *p* ≤ 0.05 was considered to be statistically significant.

## 3. Results and Discussion

### 3.1. Synthesis and Characterizations of SP

**Figure 1:**
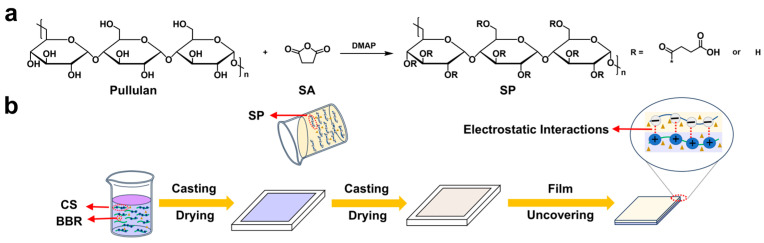
(**a**) Synthesis route of SP. (**b**) Schematic diagram of the preparation procedure of the CSSPB films.

**Figure 2:**
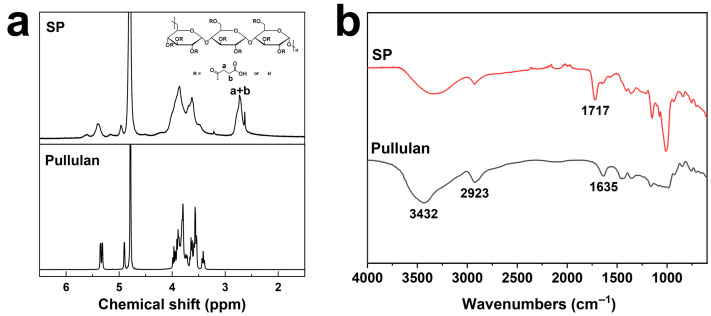
(**a**) ^1^H NMR spectra of pullulan and SP in D_2_O. (**b**) FT-IR spectra of pullulan and SP.

Under the catalysis of DMAP, a substitution reaction occurred between pullulan and SA, generating SP (Figure 1a). Figure 2 presents the ^1^H NMR and FT-IR spectra of pullulan and SP. In the ^1^H NMR spectrum of pullulan, the peaks at 4.86–5.43 and 3.33–4.09 ppm were ascribed to the anomeric and non-anomeric protons of pullulan, respectively [26]. In the ^1^H NMR spectrum of SP, the peaks at 2.5–3.0 ppm were assigned to the protons of the methylene groups that were adjacent to the carbonyl groups in SP (a, b), evidencing that pullulan was succinylated. The succinylation degree was calculated to be approximately 40% according to the peak area ratio, which was consistent with the feeding ratio. In the FT-IR spectrum of pullulan, the peaks at 3432, 2923, and 1635 cm^−1^ originated from the stretching vibrations of O–H, sp^3^ C–H, and O-C-O, respectively [26]. The absorption peak at 1717 cm^−1^ in the FT-IR spectrum of SP that was attributed to the carbonyl stretching vibration supplementarily confirmed the successful synthesis of SP.

### 3.2. Preparation, Color, Transparency, Thickness, and Micromorphology of Films

**Figure 3:**
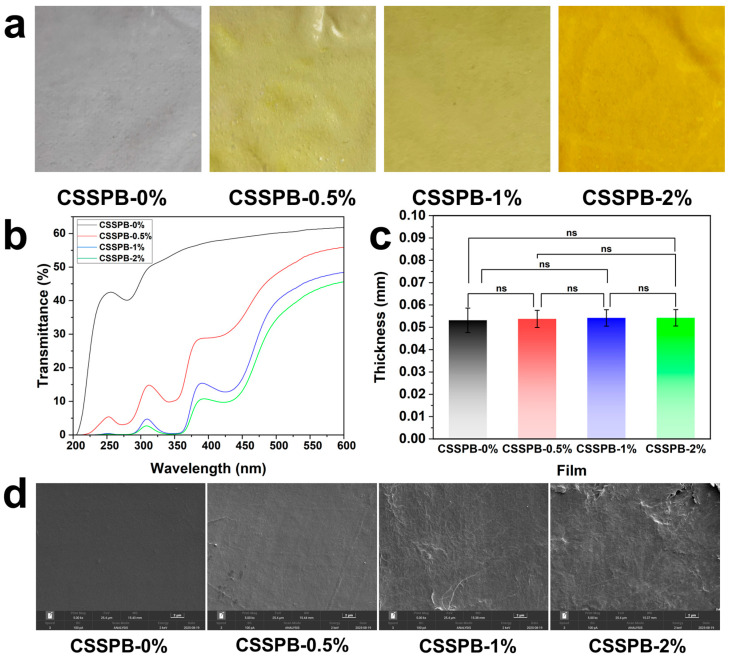
Photographs (**a**), UV–Vis spectra (**b**), thickness (*n* = 10, ns: not significant) (**c**), and SEM images (**d**) of the CSSPB-0%, CSSPB-0.5%, CSSPB-1%, and CSSPB-2% films. The scale bar in Figure 3d is 2 μm.

As depicted in Figure 1b, the CSSPB films were prepared using the layer-by-layer assembly method. The chitosan solution in the mold was dried to obtain the chitosan film, onto which the SP solution was then added. After drying, the CSSPB composite film was formed and peeled off. Electrostatic interactions were speculated to exist between the ammonium groups of chitosan and the carboxylate groups of SP. The effects of BBR addition on the color, transparency, thickness, and micromorphology of the film were studied first. As exhibited in Figure 3a, the CSSPB-0% film was colorless and transparent. After BBR was added, the films turned yellow. As the content of BBR increased, the color of the film gradually deepened and its transparency gradually decreased. The transparency of the films was further quantitatively analyzed using the UV–Vis spectra (Figure 3b). It was found that the light transmittance of the CSSPB-0% film was higher than those of the films containing BBR, and the light transmittance gradually decreased with the increase in BBR content. For instance, at a wavelength of 600 nm, the light transmittance of the CSSPB-0%, CSSPB-0.5%, CSSPB-1%, and CSSPB-2% films was 61.8%, 55.9%, 48.4%, and 45.6%, respectively. Film thickness, an important parameter of packaging films, is closely related to their mechanical properties. With the increase in BBR content, the film thickness did not change significantly, only increasing from 53 ± 5 μm for the CSSPB-0% film to 54 ± 4 μm for the CSSPB-2% film (Figure 3c). This was because the addition amount of BBR was extremely small, relative to the amount of other components in the film. The micromorphology of the films was observed by SEM (Figure 3d). With the BBR content increasing, the roughness of the film surface gradually increased. Nevertheless, all the film surfaces presented a dense structure without obvious holes and cracks, indicating that the films had good compatibility with BBR.

### 3.3. Mechanical Properties of Films

**Figure 4:**
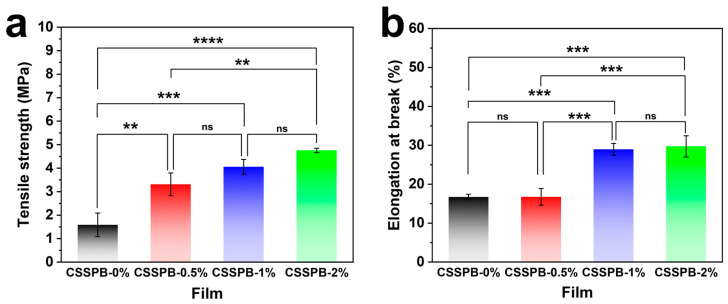
TS (**a**) and EAB (**b**) of the CSSPB-0%, CSSPB-0.5%, CSSPB-1%, and CSSPB-2% films (*n* = 3, ns: not significant, ** *p* < 0.01, *** *p* < 0.001, and **** *p* < 0.0001).

The mechanical properties of a packaging film play an important role in maintaining its integrity when it is subjected to external stress [27]. In order to evaluate the mechanical properties of the CSSPB films, their TS and EAB were determined by using a universal material testing machine (Figure 4). With an increase in BBR content, the TS of the films increased from 1.59 ± 0.50 MPa to 4.76 ± 0.09 MPa, and the EAB of them rose from 16.71 ± 0.70% to 29.71 ± 2.73%. These results suggested that the intermolecular interactions within the films were probably enhanced by the incorporation of BBR. Similar phenomena were also observed in previous studies about packaging films incorporating other natural additives, such as rambutan (*Nephelium lappaceum* L.) peel extract [28] and *Zanthoxylum limonella* essential oil [29]. The appropriate TS and EAB values in this study indicated the potential of these films for use as food packaging materials.

### 3.4. MC

**Figure 5:**
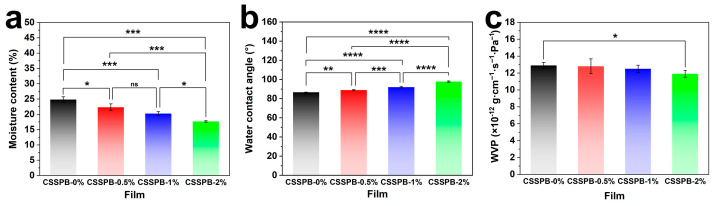
MC (**a**), WCA (**b**), and WVP (**c**) of the CSSPB-0%, CSSPB-0.5%, CSSPB-1%, and CSSPB-2% films (*n* = 3, ns: not significant, * *p* < 0.05, ** *p* < 0.01, *** *p* < 0.001, and **** *p* < 0.0001).

MC is an important factor influencing the water barrier properties of a packaging material [30], and therefore, the MC of the CSSPB films was determined. As displayed in Figure 5a, the MC of the films decreased from 24.83 ± 0.90% to 17.67 ± 0.23% with an increasing BBR content. This significant change was primarily attributed to the following reasons. The core structure of BBR consists of an isoquinoline ring and a dihydroisoquinoline ring [31], meaning that BBR possesses certain hydrophobicity. Its incorporation into the film effectively reduced the overall hydrophilicity of the film, thereby diminishing the water-holding capacity of the film.

### 3.5. WCA

WCA is a crucial indicator for evaluating the wettability of a film’s surface. A contact angle of less than 90° indicates a hydrophilic surface, while a contact angle of greater than 90° indicates a hydrophobic surface [32]. As illustrated in Figure 5b, the CSSPB-0% film exhibited a WCA of 86.7° ± 0.2°, reflecting the hydrophilicity of its surface. The incorporation of hydrophobic BBR led to an increase in the WCA of the composite films. The WCAs of both the CSSPB-1% and CSSPB-2% films surpassed 90°, and the CSSPB-2% film had the largest WCA of 97.8° ± 0.7°, suggesting the transition from the hydrophilic surface to the hydrophobic surface. The phenomena could be explained as follows: Firstly, the addition of hydrophobic BBR effectively reduced the overall hydrophilicity of the film. Secondly, the incorporation of BBR increased the surface roughness of the films (Figure 3d). The enhanced surface roughness also contributed to the improved hydrophobicity [33,34].

### 3.6. WVP

WVP is usually adopted to evaluate the water barrier properties of packaging films. Packaging films require a low WVP representing excellent water vapor barrier properties [35]. As shown in Figure 5c, the WVP value of the CSSPB-0% film was (1.29 ± 0.34) × 10^−11^ g·m^−1^·s^−1^·Pa^−1^. The incorporation of the hydrophobic BBR resulted in a decrease in the WVP of the composite films, indicating that BBR enhanced the water vapor barrier properties. When x was 2%, the WVP reached a minimum value of (1.19 ± 0.41) × 10^−11^ g·m^−1^·s^−1^·Pa^−1^. The results could be attributed to the inherent hydrophobicity of BBR itself and more tortuous paths BBR created for water vapor to transmit through the film [22]. The low WVP of the films demonstrated that they held application potential in food packaging, where moisture retention is crucial.

### 3.7. FT-IR Spectra

**Figure 6:**
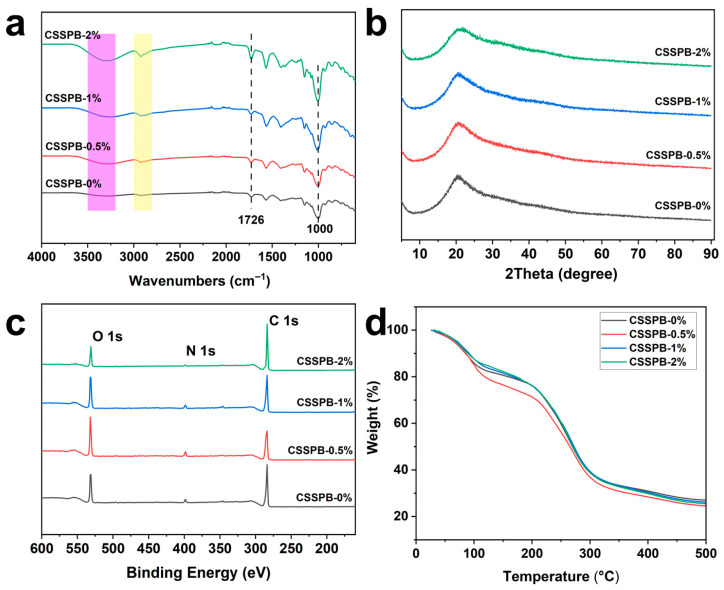
FT-IR spectra (**a**), XRD patterns (**b**), XPS spectra (**c**), and TGA curves (**d**) of the CSSPB-0%, CSSPB-0.5%, CSSPB-1%, and CSSPB-2% films.

Figure 6a presents the FT-IR spectra of the CSSPB films. In all the spectra, a broad absorption band observed in the wavenumber range of 3200 to 3500 cm^−1^ was ascribed to the stretching vibrations of hydroxyl (-OH) and amino (-NH_2_) groups [36]. The absorption peaks at 3000–2800 cm^−1^, 1726 cm^−1^, and ~1000 cm^−1^ corresponded to the stretching vibrations of sp^3^ C–H, C=O in SP, and C–O, respectively. All spectra were similar, indicating that the introduction of a very small amount of BBR did not significantly affect the chemical bonding in the film.

### 3.8. XRD

XRD was employed to investigate the microstructure of the films. All films exhibited a broad peak at 2θ ≈ 20° (Figure 6b), which might be the characteristic diffraction peaks of chitosan [37] and SP. Notably, the XRD patterns showed no significant changes after the addition of BBR. This result indicated that BBR probably did not alter the crystalline properties of the film.

### 3.9. XPS

XPS can reflect the elemental composition of a film’s surface. The XPS results of the films are displayed in Figure 6c. The peaks at 284, 398, and 531 eV corresponded to C1s, N1s, and O1s, respectively, suggesting that the CSSPB films contained carbon, nitrogen, and oxygen elements, which was consistent with the elemental composition of the raw materials.

### 3.10. TGA

As illustrated in Figure 6d, the thermogravimetric curves of the CSSPB-0.5%, CSSPB-1%, and CSSPB-2% films showed no significant differences compared to that of the CSSPB-0% film. This indicated that they possessed almost the same thermal stability. The thermal degradation processes of all the films were divided into three distinct stages. In the first stage (30–120 °C), the mass of the film gradually decreased due to the evaporation of free water, with a mass loss ratio of ~18%. The weight loss (~8%) in the second stage (120–200 °C) was mainly caused by the volatilization of glycerol and bound water. The third stage ranged from 200 to 500 °C, and the remarkable mass loss (~48%) in this stage possibly resulted from the severe degradation of both chitosan and SP. The maximum weight-loss temperature of all films was ~266 °C.

### 3.11. Antioxidant Capacities of Films

**Figure 7:**
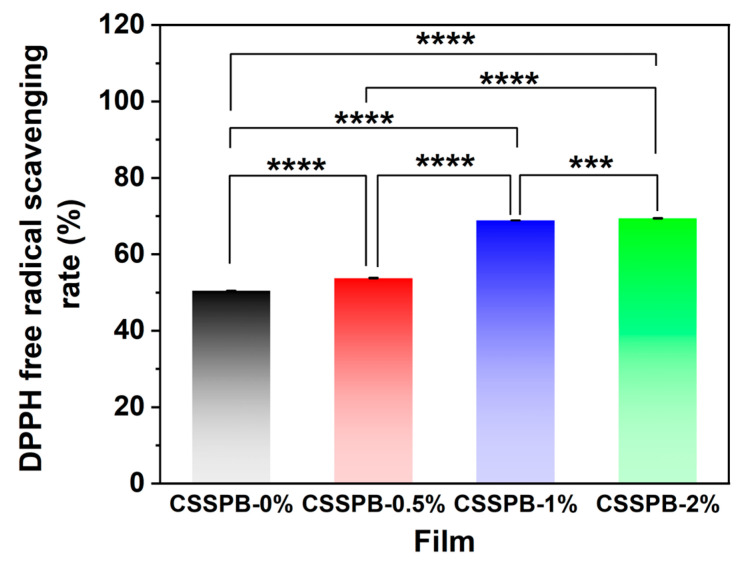
DPPH radical scavenging rates of the CSSPB-0%, CSSPB-0.5%, CSSPB-1%, and CSSPB-2% films (*n* = 3, *** *p* < 0.001, **** *p* < 0.0001).

To evaluate the films’ potential in delaying food oxidation, their antioxidant capacities were analyzed through a DPPH radical scavenging assay. As shown in Figure 7, the CSSPB-0% film exhibited a DPPH radical scavenging rate of 50.48 ± 0.06%. This could be due to the inherent antioxidant properties of chitosan itself [38]. After adding BBR, the DPPH radical scavenging capacities of the films were further enhanced (*p* < 0.0001) and showed a positive correlation with the BBR content. The maximum DPPH radical scavenging rate reached 69.39 ± 0.10%. These results indicated that the addition of BBR prominently improved the antioxidant activities of the films, which was beneficial for their application in food packaging.

### 3.12. Antifungal Activities of Films

**Figure 8:**
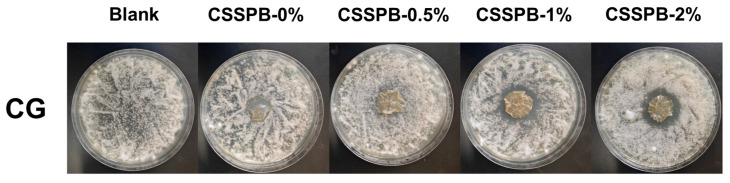
Inhibition zones of the CSSPB-0%, CSSPB-0.5%, CSSPB-1%, and CSSPB-2% films against CG.

CG is a widely distributed and highly destructive plant pathogenic fungus that can infect fruits such as citrus [39], mango [40], and pomegranate [41], leading to fruit quality deterioration and significant postharvest economic losses. Therefore, its control is of great importance. As shown in Figure 8, no obvious inhibition zones were observed for the CSSPB-0% and CSSPB-0.5% films, whereas clear inhibition zones appeared for the CSSPB-1% and CSSPB-2% films (the diameters of the inhibition zones of the CSSPB-1% and CSSPB-2% films were 2.503 ± 0.025 and 2.933 ± 0.059 cm, respectively), revealing that the CSSPB-0% and CSSPB-0.5% films had no diffusion-dependent antifungal activities, and the CSSPB-1% and CSSPB-2% films exhibited significant diffusion-dependent antifungal efficacy. The possible reason for these phenomena was that when a small amount of BBR was incorporated into the film, the release amount of BBR was too small to exert its antifungal effects. However, as the BBR content increased, the amount of BBR released from the film also increased, and thus effective antifungal properties could be imparted to the film.

### 3.13. Preservation Effects of Films on Fresh-Cut Apples

**Figure 9:**
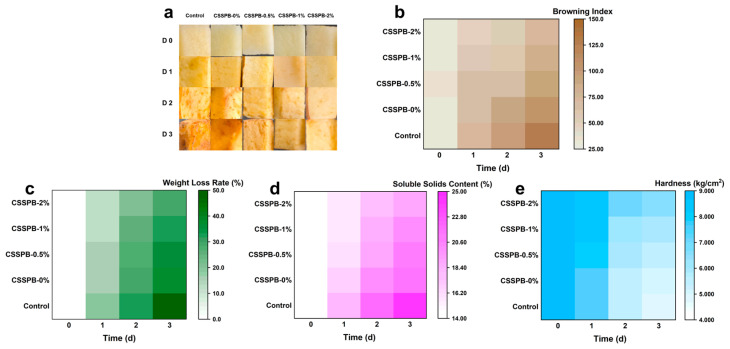
Preservation effects of the CSSPB films on fresh-cut apples during a three-day storage period. Photographs (**a**), BI values (**b**), weight loss rates (**c**), soluble solids contents (**d**), and hardness (**e**) of fresh-cut apples (*n* = 3).

To validate the practical application potential of the CSSPB films, the preservation effects of them on fresh-cut apples were systematically evaluated. Figure 9a depicts the appearances of the fresh-cut apples in the CSSPB film-treated and control groups. It was evident that the quality of all apple samples gradually declined over time, and the quality deterioration manifested as browning and wrinkling. On days 1, 2 and 3, the quality of the apples in all experimental groups was observed to be higher than that in the control group. In the film-treated groups, as the BBR content increased, the quality deterioration of the apples gradually slowed. The above observations qualitatively unveiled that the films possessed certain preservation effects.

The BI of fresh-cut apples reflects the degree of browning of them, and a higher index indicates more severe browning [27]. Figure 9b shows the BI of the apples. Over time, the BI of all of the fresh-cut apples increased. The BI of the experimental groups was smaller than that of the control group on days 1, 2, and 3, which was probably due to the inhibition of the polyphenol oxidase-mediated enzymatic browning reactions by the films with antioxidant properties. In the groups treated with the CSSPB films, with the increasing content of BBR in the film, the increase in the BI was seen to gradually slow down. This was probably because the film with a higher BBR content was in possession of stronger antioxidant capacities, and could more strongly inhibit the browning reactions.

The weight loss rate is an important indicator of the ability of postharvest fruits and vegetables to retain moisture. As exhibited in Figure 9c, with time going by, the weight loss rates of all the fresh-cut apples became higher and higher. On days 1, 2, and 3, the weight loss rate of the apples in the control group was higher than those in the film-treated groups. In the experimental groups, the apples treated by the films with a higher BBR content showed a slower increase in the weight loss rate. The phenomena could be explained as follows: The films with certain water vapor barrier properties were able to delay moisture loss. Additionally, with the content of BBR in the film increasing, the water vapor barrier properties of the film were improved, leading to the slower water loss of apples.

From Figure 9d, it could be observed that the soluble solids contents of all groups showed an increasing trend during storage. Two reasons might be responsible for this rise: First, the hydrolysis of protopectin and starch was conducive to the increase in soluble solids content [42]. Second, the water loss of fresh-cut apples could result in an increase in soluble solids concentration. On days 1, 2, and 3, the soluble solids contents of all experimental groups were found to be lower than that of the control group (*p* < 0.01), which was likely because the activities of hydrolases were inhibited by the films, and the films possessing water vapor barrier performances reduced the moisture loss of apples, thereby making the soluble solids contents relatively low. Compared with the group treated with the film with a lower BBR content, the group treated with the film with a higher BBR content showed a slower increase in the soluble solids content. The following possible reasons could be used to explain this phenomenon: BBR may be capable of inhibiting the activities of hydrolases. The higher the content of BBR in the film was, the slower the enzyme-mediated hydrolysis of protopectin and starch was. The water vapor barrier properties of the film with a higher BBR content were more excellent, and the apples packaged with it might lose moisture more slowly.

Hardness is a crucial indicator of fruit quality. The changes in hardness of the fresh-cut apples are shown in Figure 9e. The hardness of all fresh-cut apple samples decreased over time, with the control group exhibiting the most rapid hardness decline. In the film-treated groups, as the content of BBR increased, the decrease in the hardness of the fresh-cut apples slowed. These results could be rationalized as follows: The films had certain antioxidant capacities that were possibly beneficial for slowing down the hardness decrease. The film with a higher BBR content possessed stronger antioxidant properties when compared to that with a lower BBR content, which were more conducive to delaying the decline in hardness.

Based on the above experimental results, the CSSPB films were perceived to have excellent preservation effects on fresh-cut apples, and could be potentially employed in the field of food packaging.

It should be noted that during use, BBR possibly migrates from films to fruits. Considering that BBR is an active compound, this migration may have certain effects on food safety. In our future studies, the migration test and relevant discussions will be conducted.

## 4. Conclusions

In this study, chitosan, SP, and BBR were integrated to successfully develop novel biopolymer-based composite films (CSSPB), using the layer-by-layer assembly method. The physicochemical properties of the CSSPB films were systematically investigated, and the incorporation of BBR was observed to significantly influence some of the physicochemical properties of the film. For example, with an increasing BBR content, the TS increased from 1.59 MPa to 4.76 MPa, the EAB rose from 16.71% to 29.71%, the hydrophobicity was improved with the WCA increasing from 86.7° to 97.8°, and the antioxidant capacities were enhanced, with the CSSPB-2% film showing the maximum DPPH radical scavenging rate of 69.39%. Notably, when the x value was ≥1%, the film exhibited observable antifungal activities against the common plant pathogenic fungus CG. In the preservation experiments, the CSSPB films were found to be capable of effectively delaying the quality deterioration of the fresh-cut apples. We believe that the CSSPB films in this work are a promising food packaging material, and will inspire the design and development of new biopolymer-based packaging films.

## Data Availability

The original contributions presented in the study are included in the article. Further inquiries can be directed to the corresponding authors.
